# Russeting in ‘Apple’ Mango: Triggers and Mechanisms

**DOI:** 10.3390/plants9070898

**Published:** 2020-07-16

**Authors:** Thomas O. Athoo, Andreas Winkler, Moritz Knoche

**Affiliations:** Institute of Horticultural Production Systems, Leibniz University Hannover, Herrenhäuser Straße 2, 30419 Hannover, Germany; thomasathoo@gmail.com (T.O.A.); andreas.winkler@obst.uni-hannover.de (A.W.)

**Keywords:** *Mangifera indica*, skin, periderm, cuticle, epidermis, lenticel

## Abstract

Russeting is an important surface disorder of many fruitcrop species. The mango cultivar ‘Apple’ is especially susceptible to russeting. Russeting compromises both fruit appearance and postharvest performance. The objective was to identify factors, mechanisms, and consequences of russeting in ‘Apple’ mango. Russeting was quantified on excised peels using image analysis and a categorical rating scheme. Water vapour loss was determined gravimetrically. The percentage of the skin area exhibiting russet increased during development. Russet began at lenticels then spread across the surface, ultimately forming a network of rough, brown patches over the skin. Cross-sections revealed stacks of phellem cells, typical of a periderm. Russet was more severe on the dorsal surface of the fruit than on the ventral and more for fruit in the upper part of the canopy than in the lower. Russet differed markedly across orchards sites of different climates. Russet was positively correlated with altitude, the number of rainy days, and the number of cold nights but negatively correlated with minimum, maximum, and mean daily temperatures, dew point temperature, and heat sum. Russeted fruit had higher transpiration rates than non-russeted fruits and higher skin permeance to water vapour. Russet in ‘Apple’ mango is due to periderm formation that is initiated at lenticels. Growing conditions conducive for surface wetness exacerbate russeting.

## 1. Introduction

Russeting is a surface disorder of many fruitcrop species worldwide. In botanical terms, russet represents the formation of a periderm [1] comprising three layers: a phellogen (meristematic) that gives rise to a phelloderm (to the inside) and a phellem (to the outside). The phellem comprises stacks of cork cells. It is their suberised cell walls that are responsible for the rough, brown appearance of a russeted fruit skin. This appearance is generally unattractive to the consumer [2]. Russet therefore compromises the visual quality of a fruit and thus excludes it from the high-value export markets. Russet is also associated with increased postharvest water loss, which further compromises postharvest performance [3]. This requires fruit cartons to be “overpacked” if they are to reach the end consumer at the pre-specified weight. For both these reasons, russeting has serious economic consequences for the grower.

*Malus* apple is a prominent example of a susceptible fruit crop. Most information on the ontogeny of russet is available for this species. In apple, russet is preceded by the formation of microcracks in the cuticle [4,5]. Surface wetness [6,7], agrichemicals [8,9], and pests and diseases such as mites [10], epiphytic fungi [11], and bacteria [12] are all factors aggravating russeting. A periderm forms, presumably in the hypodermal cell layers [13,14]. The cuticle and the epidermis dry out and slough off as the phellem develops. The brownish cork cells are then revealed on the fruit surface. 

The ‘Apple’ mango is a valuable mango cultivar in the Kenyan market. It has excellent texture and flavour. Unfortunately, ‘Apple’ mango is also highly susceptible to a skin disorder that bears similarity to the well-known russet of many apple and pear cultivars. To our knowledge, there is no information available on this disorder in mango. 

The objective of this study was (1) to identify whether the “russet” of ‘Apple’ mango is caused by a periderm formation and (2) to identify the agronomic and the environmental factors affecting the incidence and severity of “russeting” in this cultivar.

## 2. Results

**Figure 1:**
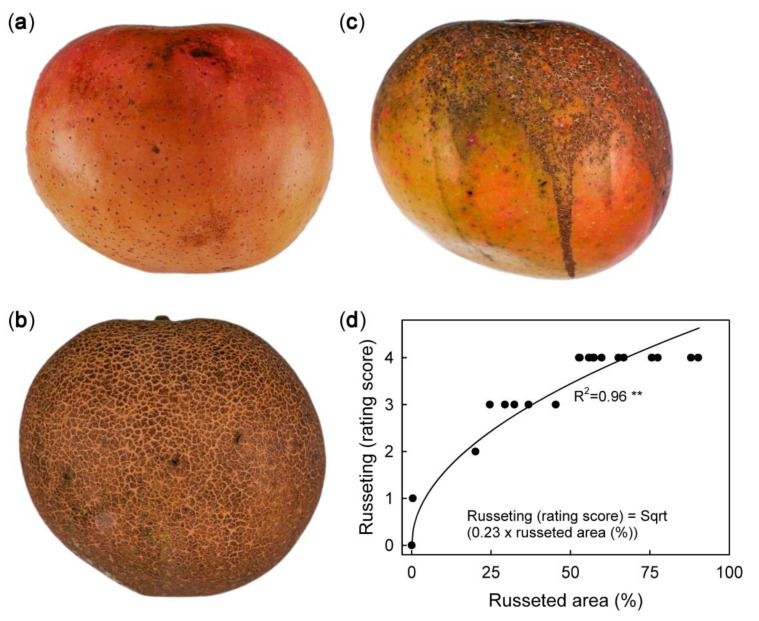
Macroscope view of mature ‘Apple’ mango without (**a, score 0**), moderate (**b, score 2**), and extreme (**c, score 4**) russet symptoms. (**d**): Plot of russeting (rating score) against percentage area affected by russet (image analysis). Each fruit was rated visually prior to image analysis. The number of observations was 18.

Russet severity in ‘Apple’ mango was non-uniform within a tree and across an orchard. The severity of russet in the same orchard ranged from non-russeted (Figure 1a, score 0) to moderate (Figure 1c, score 2) to extreme (Figure 1b, score 4). The russet scores of the rating scheme used to quantify russet were closely correlated to the actual russeted surface area measured by image analysis (Figure 1d).

**Figure 2:**
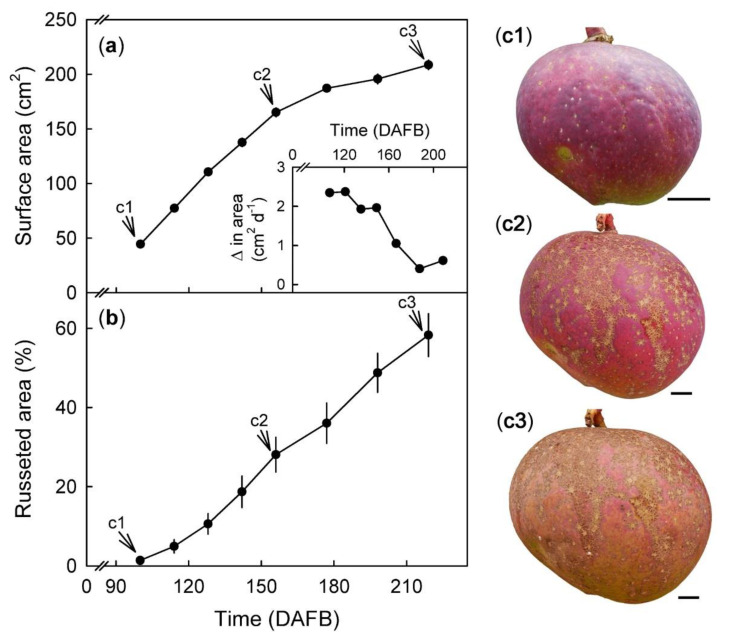
Change in fruit surface area (cm^2^) and rate of surface expansion (**a** and **a inset**) with time (days after full bloom, DAFB). Percent of skin with russet in developing fruit (**b**) calculated from a defined area of the fruit cheek. The same fruit was photographed at 100, 156, and 216 DAFB (see arrows). Pictorial representation of russet progression in a developing ‘Apple’ mango fruit (**c1**–**c3**). Scale bar is 10 mm. Data represent means ± SE of 19 replicates.

Fruit surface area increased with time (Figure 2a). The growth rate in surface area was at a maximum of 2.3 cm^2^ day^−1^ at about 114 days after full bloom (DAFB) and decreased continuously thereafter (Figure 2a inset). The percentage of the surface area of the skin exhibiting russet increased with time throughout development (Figure 2b,c). 

**Figure 3:**
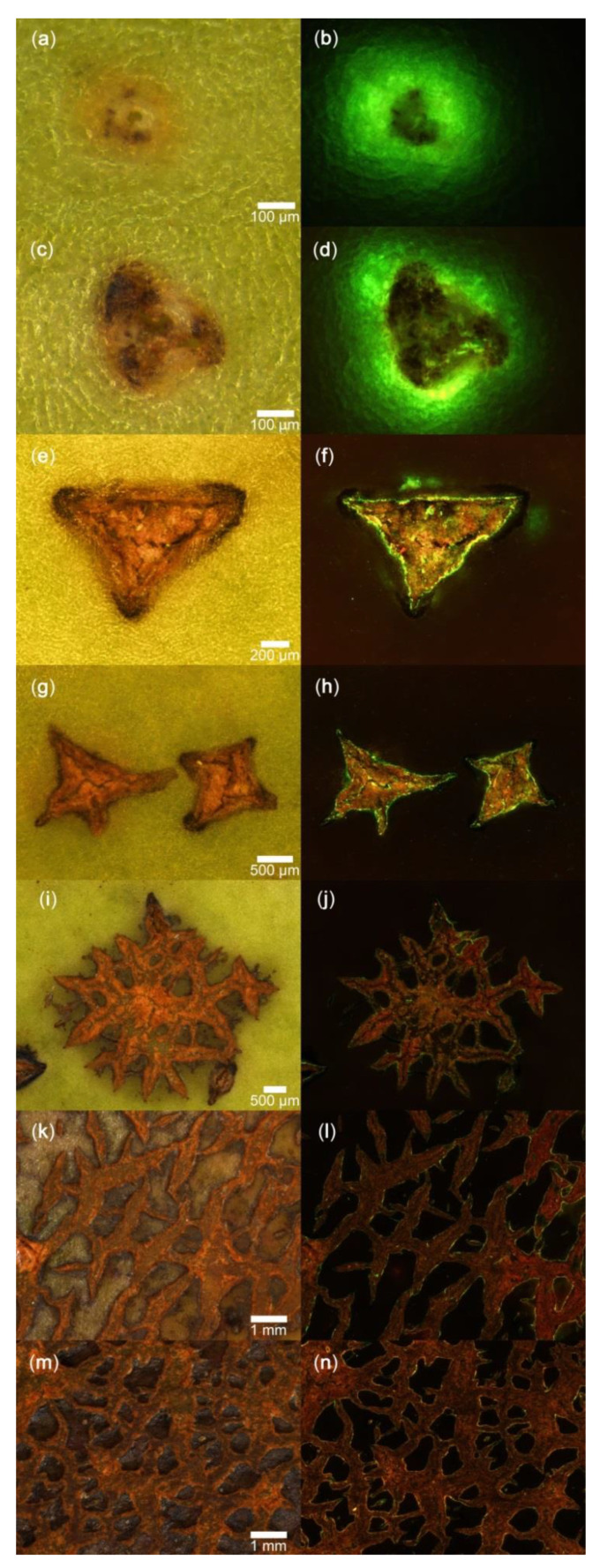
Microscopic view of ‘Apple’ mango skin infiltrated with acridine orange dye when viewed with a binocular microscope under natural (**a**,**c**,**e**,**g**,**i**,**k**,**m**) or fluorescent light (**b**,**d**,**f**,**h**,**j**,**l**,**n**). The scale bars refer to the corresponding pairs of images.

Microscopic inspection of the fruit surface following labelling with acridine orange revealed that initial cracking always began at a lenticel (Figure 3a–d). Lenticels ruptured and developed into (usually) three- or four-pointed star- or triangular-shaped short cracks. These were filled with periderm (Figure 3e–h). These stellate cracks enlarged and merged as cracks propagated and development progressed. They eventually formed islands of crack networks. These islands later expanded and merged. The end result was an extensive network of rough, brown patches. Occasionally, these patches extended over the entire fruit surface (Figure 3i–n). Only during the initial stages of cracking was there significant infiltration of acridine orange at the lenticels (Figure 3b,d). There was essentially no infiltration after the periderm had developed (Figure 3h,j,l,n). 

**Figure 4:**
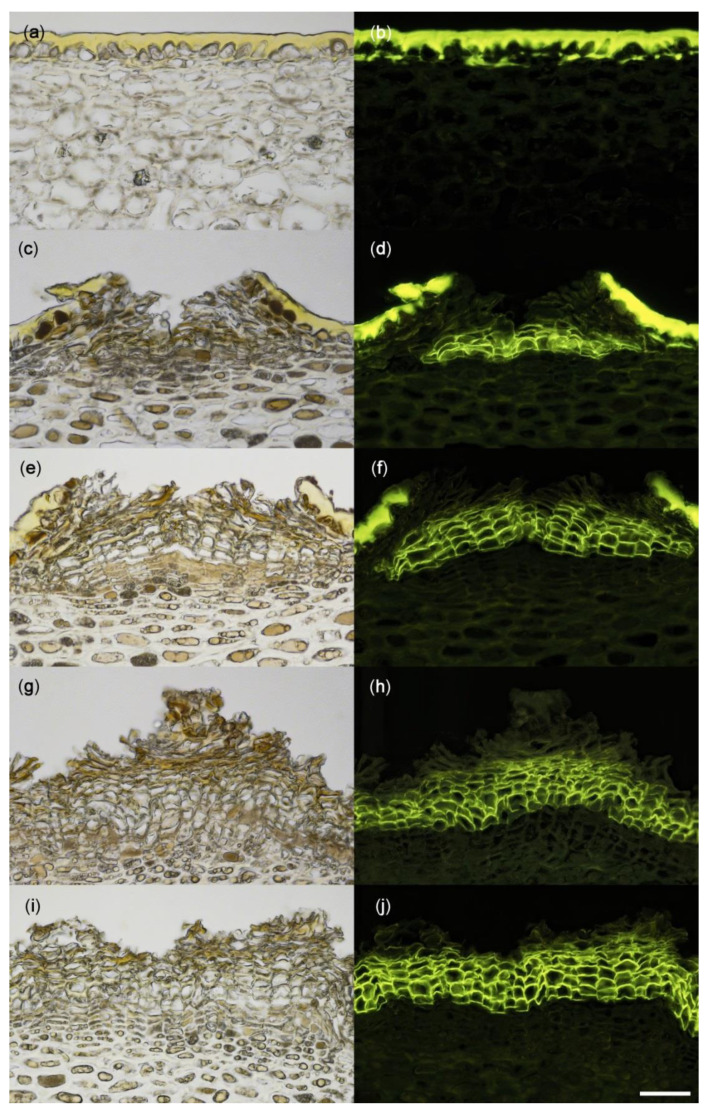
Cross-sectional microscope images of a non-russeted (**a**,**b**) and russeted (**c**–**j**) skin of ‘Apple’ mango viewed under incident white (**a**,**c**,**e**,**g**,**i**) or fluorescent light (**b**,**d**,**f**,**h**,**j**) following staining with fluorol yellow dye. Scale bar is 50 µm.

The non-russeted fruit skin had an intact cuticle, epidermis, and hypodermis (Figure 4a,b). There was no cuticle or epidermis on the lenticels (Figure 4c–j). The pore of the lenticel was filled with stacks of thick-walled cells varying from three layers (initial stage of russeting) to more than five (extreme russeting) (Figure 4c–j). The walls of these cells fluoresced following staining with fluorol yellow. This identified them as the suberised (corky) walls of a typical periderm (Figure 4d,f,h,j).

For any particular fruit, russet severity decreased along the stem/apex axis. Russet was most severe at the stem end and least severe at the apex (Table 1). There were no significant differences in the severity of russeting between the blushed and the non-blushed sides of a fruit (Table 1).

Russeting was significantly less severe in the ventral region than in the dorsal region of a fruit (Table 2). This effect was consistent across the three orchard sites, which differed significantly in overall russeting severity. Russeting was consistently most severe in Kaiti, followed by Mumbuni, and was least severe in Yeemulwa (Table 2).

Within the canopy, there were no significant differences in russeting between peripheral (exposed) or central (shaded) fruits on a tree. Again, fruit from the Kaiti site had the highest incidence of russeting followed by Mumbuni and Yeemulwa (Table 3).

There was significant interaction between the orchard site and the position of the fruit within the tree. Across all sites, fruits located in the top of the canopy were more russeted than those in the middle or the bottom parts of the canopy (Table 4).

There was no significant effect of the geographical orientation of the fruit (aspect) in the canopy on russeting. Fruits exposed to north, south, east, and west all showed similar russeting across the three sites (Table 5).

**Figure 5:**
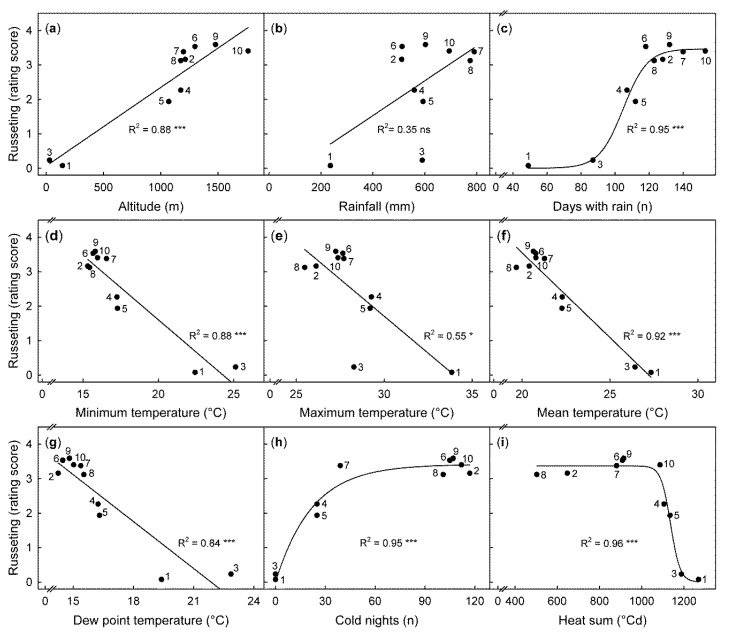
Relationship between climatic variables and average russeting (rating score) determined for the cumulative period of fruit maturity at ten locations in Kenya. The ten locations were: Garissa (1), Chepsigot (2), Malindi (3), Mumbuni (4), Yeemulwa (5), Kaiti (6), Kasafari (7), Kambirwa (8), Thika (9), and Machakos (10) situated at different altitudes. (**a**) The climatic variables include: rainfall amount (**b**), days with rainfall (**c**), relative humidity (**d**), minimum, maximum, and mean daily temperatures (**e**, **f**, and **g**, respectively). Cold nights (**h**) correspond to the number of days when the minimum temperature fell below the base temperature of 16 °C. Heat sum (**i**) was calculated based on a base temperature of 16 °C. Russeting was quantified using a five-score rating scheme: score 0: 0% of the fruit surface area russeted; score 1: 1–10% russeted area; score 2: 11–25% russeted area; score 3: 26–50% russeted area; and score 4: 51–100% russeted area. Data points represent means of 210 fruit per site.

Russeting differed markedly between the ten sites across Kenya. Russeting was highest in Thika, Kaiti, Machakos, and Kasafari and lowest in Garissa and Malindi (Table 6). These sites differed markedly in climate. Analysis of potential relationships between climatic parameters and russet severity revealed the following relationships; highly significant, linear, positive relationships were obtained between altitude and russeting, i.e., there was more russeting at higher altitudes. Furthermore, russeting was significantly correlated with the number of rainy days but not with either the amount of rainfall (mm) or the relative humidity (%). Russeting was negatively correlated with minimum, maximum, and mean daily temperatures, and dew point temperatures. Positive relationships were observed for the number of cold nights, a negative sigmoidal one for the heat sum (Figure 5).

There was little difference in the correlation coefficients between russet and the various weather variables during the first, the middle, and the later parts of the growing season. The only remarkable exception was the relationship of russet to the number of cold nights. Here, cold nights during early fruit development were particularly associated with increased russeting (Table 7).

**Figure 6:**
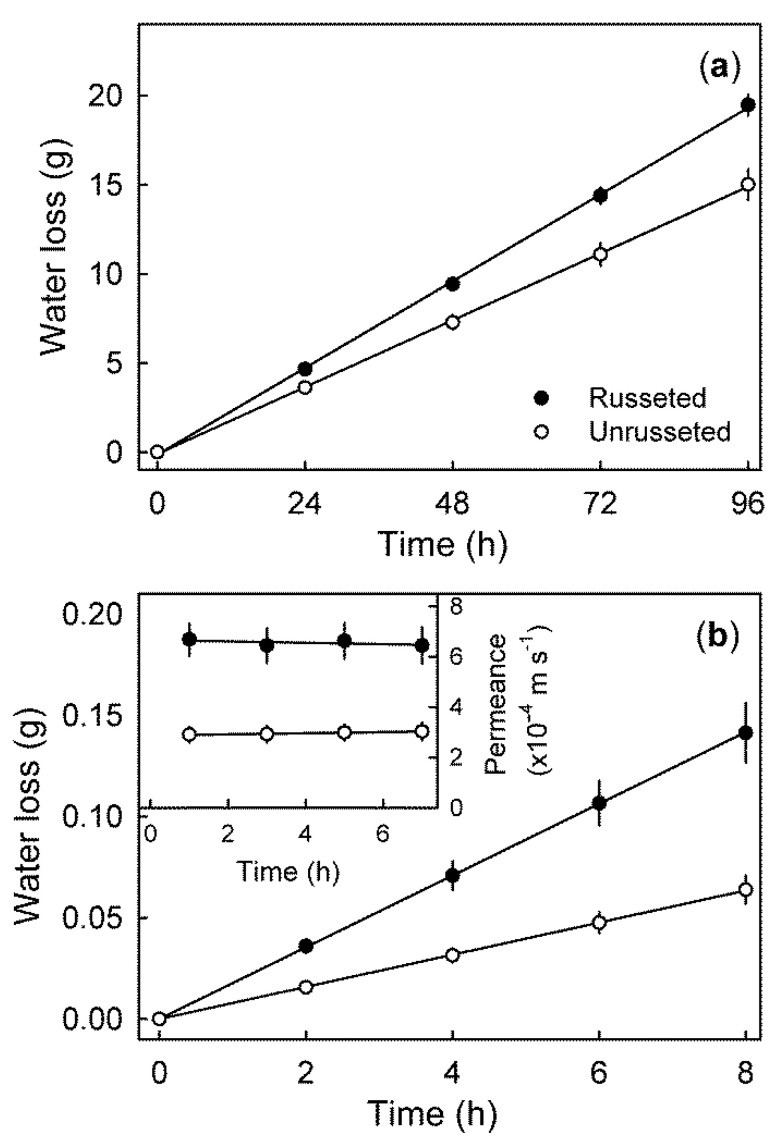
Time course of transpiration by whole fruits with extreme (>50%) (russeted) and with minimal (<25%) russet (not russeted) (**a**) and through epidermal sections (ES) excised from the cheek of mature ‘Apple’ mango fruit with and without russeting (**b**). Permeance of water vapour diffusion through the ES (**b inset**) was calculated under conditions of steady state water loss. Data represent means ± SE of a minimum of 10 replicates.

Transpiration increased linearly with time. Russeted fruit had significantly higher rates of transpiration compared to non-russeted fruits (Figure 6a). The epidermal sections (ES) from russeted skin also exhibited higher transpiration compared to non-russeted ES (Figure 6b). Permeance to water loss was constant with time but higher in russeted ES compared to control (Figure 6b inset).

## 3. Discussion

The most important findings in our study were:

(1) Russet in ‘Apple’ mango involves the formation of a periderm.
(2) Russet begins at the lenticels and from there spreads over the fruit surface.
(3) Rainy days and low night temperatures are especially conducive to russeting.

### 3.1. Russet in ‘Apple’ Mango Involves Formation of a Periderm

Russet in ‘Apple’ mango is similar to russet phenomena in other fruitcrop species, such as *Malus* apple [15,16], pear [17], citrus [18], grape [8], and melon [19]. This conclusion is based on the following arguments: (1) the appearance of the disorder in ‘Apple’ mango with rough brownish irregular patches surrounded by interconnected light brownish cracks is identical to that previously described for *Malus* apple [7]. (2) Cross-sections of the skin of ‘Apple’ mango identified stacks of suberised cells typical for a phellem produced by a phellogen as found in potatoes [20], *Malus* apple [3,13], reticulated melon [19], and grape [8]. The periderm of ‘Apple’ mango was stained with fluorol yellow, such as that of *Malus* apple [3]. (3) Russeting in ‘Apple’ mango increased during development. Russet symptoms began during early fruit development and became progressively more severe. The number of phellem layers in these stacks increased continuously. Similar observations were made in *Malus* apple, pear, and melons [15,19,21]. However, the maximum rate of increase in russet was reached later in developing ‘Apple’ mango compared with *Malus* apple or pear [15,21], where early fruit development is considered the most susceptible phase [22]. (4) As discussed below, surface wetness aggravates russeting in ‘Apple’ mango as it does in *Malus* apple [23,24]. (5) The formation of russet in ‘Apple’ mango is consistent with a repair process for cracks in the skin [16,25]. While some infiltration of lenticels was observed before the onset of russeting, there was practically no infiltration of the periderm during the later stages of russet development. Thus, formation of the periderm in ‘Apple’ mango would seem to perform the same function as that in *Malus* apple. It restores the barrier function of an impaired cuticle, thereby restoring, in part, the low water vapour permeance of the skin [3]. These arguments show that russeting in ‘Apple’ mango is similar to russeting in other fruitcrop species. It involves the formation of a classical periderm.

### 3.2. Russeting Begins at Lenticels and Then Spreads over the Surface

Lenticels are the sites where russeting is initiated in ‘Apple’ mango. The ontogeny of russet formation reveals an initial, usually stellate, crack in the centre of a lenticel. The crack then propagates across the fruit surface, merges, and thus comes to form a network of periderm that continue to spread over the enlarging fruit surface. In mango, lenticels develop under ruptured stomata [26,27]. This observation holds also for *Malus* apple [28,29]. In *Malus* apple, lenticels are often the source of multiple microcracks [30]. Microcracks in the cuticle are the first visible symptoms of russeting [4,5]. Whether this applies also to ‘Apple’ mango is not currently known. Growth strain is the driver for skin failure. Supporting evidence for a role of growth strain in russeting of ‘Apple’ mango comes from the observation that the ventral region was less russeted than the dorsal region. Compared to the ventral region, the dorsal region has a larger curvature and a larger strain rate, as indexed by a lower density of lenticels (Athoo, personal observation). Similar relationships have been reported for pear. In the latter, the cheek has a higher growth rate and, hence, a more rapid strain rate and thus is more prone to russeting than the neck [21].

Several hypotheses may account for lenticels being the sites of russet initiation in ‘Apple’ mango: (1) lenticels serve as stress concentrators, as demonstrated for the grape skin [31]. According to [31], lenticels of grapes represent a rigid structure embedded in an extensible skin. When strained, the lack of extensibility of the lenticel must be compensated for by a larger extension of the skin surrounding the lenticel. This causes stress concentrations and microcracking in the cuticle surrounding lenticels. (2) Alternatively, lenticels of ‘Apple’ mango may be structurally weaker and less rigid than the surrounding skin. When strained, the lenticel must make up for the lower extension of the surrounding skin. A lower rigidity may result from a loose, irregular arrangement of cells with large intercellular spaces in mango [27]. This may imply a lower tensile force to tear them. Here, one would expect microcracks to be associated with lenticels, as was observed in ‘Apple’ mango. Interestingly, microcracks that formed in plum were almost all associated with stomata, the usual precursors of lenticels [32]. (3) Lastly, lenticels were infiltrated by aqueous acridine orange during early development, indicating high permeability, even for liquid water. This could expose underlying cells to moisture, causing bursting of some cells or cell wall swelling, which in turn may decrease cell–cell adhesion. This sequence of events lead to cracking in sweet cherry fruit skin [33]. It is worth noting that the infiltration with dye solution was limited to the early development of a lenticel. In subsequent stages, lenticels were sufficiently suberised (lipophilic), thus they presented a formidable barrier to water vapour diffusion from the fruit and also for viscous water flow into the fruit. At present, it is not known which of the above two (opposing) hypotheses accounts for the periderm formation in ‘Apple’ mango.

### 3.3. Rainy Days and Low Night Temperatures Are Conducive to Russeting

Our study reveals surprisingly close relationships between a range of environmental variables and the extent of russet in ‘Apple’ mango in different agroecological regions across Kenya. Apparently, conditions conducive to surface wetness aggravate russeting. Higher elevations, more rainy days, decreasing night temperature, low dew point temperatures, and increasing numbers of cold nights all aggravate russeting. Our observations are consistent with the finding that exposure to moisture causes russeting in *Malus* apple [23]. The moisture-induced russeting probably resulted from moisture-induced microcracking. That surface wetness induces microcracks in the cuticle has been demonstrated for *Malus* apple [6,7,23], sweet cherry [34], and grapes [35]. Thus, we expected microcracks also to form when the skin of ‘Apple’ mango was exposed to moisture.

We do not have an explanation for the lack of a significant relationship between rainfall amount or relative humidity and russet. This may have been an artefact resulting from the confounding effects of temperature and humidity. The hot coastal region of Kenya (Malindi) also has high rainfall. Yet, fruit in this region was only marginally russeted. High temperatures and higher wind speeds in coastal areas make long periods of wetness duration less likely.

The mechanistic basis for moisture induced russeting is not clear. Knoche and Peschel [34] suggested changes in the mechanical properties of the cuticle due to hydration. A hydrated cuticle generally has a lower modulus of elasticity and a lower fracture force [34,36,37]. Additionally, hydration causes cell wall swelling, and this may decrease cell–cell adhesion, as demonstrated for the sweet cherry fruit skin [33,38]. Both findings increase the likelihood of fracture of a hydrated, strained cuticle. The microcracks formed then trigger periderm formation.

### 3.4. Conclusion

Our results provide evidence that the surface disorder of russet in ‘Apple’ mango is due to periderm formation initiated at lenticels. Growth strains then cause the periderm to spread over the fruit surface. Close relationships between the incidence of russet of ‘Apple’ mango grown at ten different sites in Kenya and the climatic conditions at the different sites indicate that conditions conducive for surface wetness clearly stimulate russeting. This is consistent with moisture-induced microcracking in the cuticle reported for many fruitcrop species. The resulting periderm partially restores the barrier function of the skin of ‘Apple’ mango. However, the permeance remains at a significantly elevated level, and this increases postharvest moisture loss of russeted ‘Apple’ mango. Whether or not developing ‘Apple’ mango is also more susceptible to fungal infections merits further investigation.

## 4. Materials and Methods

### 4.1. Plant Materials

Mature and immature ‘Apple’ mango (*Mangifera indica* L.) fruit grafted on local seedling rootstocks were harvested or observed in situ in several commercial orchards across Kenya. The sites selected and their geographical coordinates are: Chepsigot (0°31′ N, 35°34′ E), Garissa (0°26′ S, 39°37′ E), Kaiti (1°45′ S, 37°28′ E), Kambirwa (0°44′ S, 37°12′ E), Kasafari (0°28′ S, 37°40′ E), Machakos (1°26′ S, 37°13′ E), Malindi (3°14′ S, 40°05′ E), Mumbuni (1°50′ S, 37°36′ E), Thika (1°01′ S, 37°06′ E), and Yeemulwa (1°53′ S, 37°47′ E). Fruits were grown conventionally using recommended integrated crop management programmes. Unless otherwise specified, fruits were harvested at commercial maturity and processed within two days.

### 4.2. Quantifying Russeting

To quantify russeting, fruits were peeled, and the peels were flattened on a glass plate. Russeted areas were painted with blue acrylic paint using a soft hair brush to enhance contrast. The flattened peels were photographed under standardised conditions with a digital camera (Lumix DMC-G80; Panasonic Corporation, Osaka, Japan) fitted with a macro lens (Olympus M. Zuiko Digital 60 mm; Olympus Corporation, Tokyo, Japan). A ruler was included in each image for scaling. Total fruit surface area and the areas with and without russet were quantified using image processing software (ImageJ 1.52P; National Health Institute, Bethesda, MD, USA). This method provided a precise and objective assessment of the severity of russet. For routine analyses, a five-score rating scheme was developed. Scores were 0 for 0% of the fruit surface area russeted, 1 for 1–10% of russeted area, 2 for 11–25% of russeted area, 3 for 26–50% of russeted area, and 4 for 51–100% of russeted area.

Russeted fruit surfaces were also examined by light microscopy. Microscopic cracks (“microcracks”) on the fruit surface were identified following immersion of whole fruits in 0.1% (*w/w*) aqueous acridine orange (Carl Roth, Karlsruhe, Germany) for 10 min. Fruits were then rinsed with deionised water (30 s) and blotted dry using soft tissue paper. The fruit surface was then inspected under incident white and UV light using a fluorescence binocular microscope (Leica MZ10F with filter GFP plus 480–440 nm excitation, ≥510 nm emission; Leica Microsystems GmbH, Wetzlar, Germany). Calibrated digital photographs were taken (Olympus DP71; Olympus Europa Holding GmbH, Hamburg, Germany) and then analysed using image processing software (cellSens version 1.7.1.; Olympus).

### 4.3. Histology

Tissue blocks (5 × 2 mm) of the skin of mature fruit were excised using a razor blade and fixed in Karnovsky solution [39] until use. The fruit were selected to express a range of severities of russet. Prior to sectioning, the blocks were rinsed with deionised water and placed in 70% ethanol in plastic cassettes overnight (PrintMate biopsy Cassetes; Thermo Fisher Scientific, Kalamazoo, MI, USA). Samples were embedded and dehydrated as described before [40]. Briefly, tissue blocks were dehydrated in an ascending series of alcohol (70, 80, 90, and 96% *v/v* ethanol and 100% isopropanol). Thereafter, the blocks were dipped in xylol and then in a 1:1 *v/v* paraffin-xylol mixture before embedding in hot paraffin wax. The embedded tissue blocks were then cooled on ice and stored at 4 °C until use. Sections (10 μm thick) were cut with a microtome (Zeiss Hyrax M55; Carl Zeiss MicroImaging, Jena, Germany). The sections were relaxed on the surface of a warm water bath (40 °C), mounted on glass slides, and dried overnight at 40 °C. To remove the paraffin, the sections were washed in xylol, then rehydrated in aqueous ethanol solutions of decreasing concentration (96–60% *v/v*) and finally in deionised water. Staining was done for 60 min using 0.005% (*w/v*) fluorol yellow 088 (Santa Cruz Biotechnology, Dallas, TX, USA) dissolved in 50% *w/v* PEG 4000 (SERVA Electrophoresis, Heidelberg, Germany) and 45% *v/v* glycerol. The sections were rinsed with deionised water and viewed under incident bright and incident fluorescent light (filter module U-MWU 330–385 nm excitation wavelength, ≥420 nm emission wavelength; Olympus) using a fluorescence microscope (BX-60; Olympus). Calibrated images were taken (DP 73; Olympus).

### 4.4. Experiments

#### 4.4.1. Developmental Time Course

The developmental time courses of fruit growth and russeting were established. Five fruitlets per tree from a total of five trees were selected and tagged in a commercial orchard in Machakos County. Fruit were photographed (Lumix DMC-G80; Panasonic) every 14 to 21 days between 100 to 219 days after full bloom (DAFB). A ruler was included in each photograph for calibration. Fruit length and two orthogonal equatorial diameters were measured by image analysis (ImageJ 1.52P; National Health Institute). Fruit surface area was calculated from mean diameter assuming the fruit shape of a sphere as a first approximation ($A=4\pi r^{2}$). The rate of increase in surface area (cm^2^ d^−1^) was calculated as the increase in surface area in a time interval divided by the duration of the interval. The russeted area was estimated from the percentage of russeted area on virtual circular epidermal sections of about 2.5 to 3.0 cm diameter of the cheek. This region exhibited minimum curvature, and the skin section was approximately planar. The russeted area was quantified by image analysis (ImageJ 1.52P; National Health Institute). The mass of 15 fruits picked at random on each sampling date was determined.

#### 4.4.2. Effect of Region of the Fruit Surface

**Figure 7:**
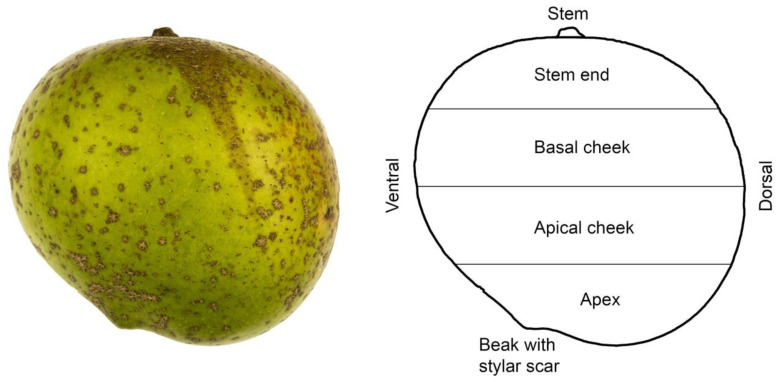
Photograph of ‘Apple’ mango and sketch illustrating the nomenclature used to describe regions of the fruit surface [41].

To quantify the distribution of russet along the stem/apex axis of the fruit, fresh fruit were selected with russet incidence ranging from a score of 1 to 4. The fruits were sliced into four regions perpendicular to the stem/apex axis representing the stem end, the basal cheek, the apical cheek, and the apex (Figure 7). These regions were further partitioned into the ventral and the dorsal sides or the blushed and the non-blushed sides of the fruit. The ventral side refers to the cheek on the side of the stylar scar, the dorsal side to the opposite side. The blushed side refers to the side that was exposed to sunlight and developed a red/orange colouration. The non-blushed side refers to the side opposite the blushed side. Russeting was quantified in the different regions on a total of 18 fruit using image analysis (ImageJ 1.52P; National Health Institute).

Potential differences in the severity of russeting between the ventral and the dorsal sides of the same fruit were investigated. A minimum of 200 fruits per site were rated for russeting using the rating scheme described above. The analysis was carried out at Kaiti (1468 m), Mumbuni (1240 m) and Yeemulwa (1013 m). These sites were selected because they differ significantly in elevation.

#### 4.4.3. Distribution of Russeted Fruit within the Canopy

The distribution of russeted fruit within the canopy was determined on a minimum of 200 fruits per site using the rating scheme described above. The three sites were Kaiti, Mumbuni, and Yeemulwa. Mature fruits located either in the periphery of the canopy (exposed) or in the centre of the canopy (shaded) were inspected and rated. In a second experiment, the role of the height of the fruit in the canopy was investigated. Here, fruits from the top (over 2 m above the ground), the middle (1–2 m), and the bottom (0.5–1 m) layers of the canopy were rated. Fruits below 0.5 m from the ground were excluded.

To test the effect of tree orientation, fruits exposed from north, south, east, and west quadrants of the canopy were selected, inspected, and rated for russeting, as described above.

#### 4.4.4. Effect of Orchard Site on Russeting

To establish potential relationships between russeting and climatic conditions, mature fruits were selected from ten different sites. Fruit were inspected and rated for russeting using the scheme described above. The sites were: Thika, Machakos, Yeemulwa, Mumbuni, Kaiti, Malindi, Garissa, Kasafari, Kambirwa, and Chepsigot. The altitude of these sites was determined using Google Earth (Version 9.3.109.1, Google LLC, Mountain View, CA, USA). Daily rainfall, relative humidity, and daily minimum, mean, and maximum temperatures during the growing season were obtained from the website of the NASA Langley Research Center (LaRC) POWER Project funded through the NASA Earth Science/Applied Science Program (NASA Langley Research Center, Hampton, VA, USA). Potential relationships between russet scores and climatic variables were investigated using correlation and regression analyses. Heat sums were calculated using a base temperature of 16 °C [42].

#### 4.4.5. Transpiration

The effect of russet on postharvest water vapour loss was investigated on intact mature fruits with and without russet. Since it was impossible to identify a sufficient number of fruits with 0% russet (score 0), fruits with less than 10% russet (score 1) were included in the category of non-russeted fruit. The russeted category had a russet score of 3–4. Fruit heights and diameters were measured using digital callipers (CD-30PK; Mitutoyo, Kawasaki/Kanagawa, Japan). The stem end was sealed with silicone rubber (Dow Corning SE 9186; Dow Corning Corp, Midland, MI, USA). Fruits were incubated in a polyethylene (PE) box containing a saturated slurry of NaCl generating a relative humidity of about 75% (equivalent to 14.6 g m^−3^ at 22 °C; [43]). Fruit were weighed (Sartorius Pro 32/34F micro scales, Sartorious AG, Göttingen, Germany) every 24 h for 96 h. The rate of water loss (F, g h^−1^) was calculated from the slope of a linear regression line fitted through a plot of water loss (g) against time (h) on an individual fruit basis.

Epidermal skin segments (ES) were excised from russeted and non-russeted regions of the fruit surface using a biopsy punch (10 mm diameter) (Kai Europe, Solingen, Germany). The cut surface of the ES was blotted dry. The ES were mounted in custom made stainless steel diffusion cells using high-vacuum grease (Korasilon-Paste; Kurt Obermeier GmbH & Co. KG, Bad Berleburg, Germany) such that the outer surfaces with the cuticle were exposed in the 7 mm orifices. Diffusion cells were filled with deionised water through a hole in the base. This hole and the gap between the bottom and the top of the diffusion cells were subsequently sealed using clear transparent tape (Tesa Film^®^; Tesa-Werke Offenburg GmbH, Offenburg, Germany). The cells were turned upside down and left overnight to equilibrate. The next morning, the diffusion cells were weighed and placed in a PE box above dry silica gel. The cells were repeatedly weighed on a digital analytical balance (Pioneer TM, OHAUS Europe GmbH, Nänikon, Switzerland) every 2 h for 8 h. The rate of water loss was calculated as described above. The permeance (P, m s^−1^) of the ES to water vapour loss was calculated from P = F/(A · ΔC). In this equation, A (m^2^) is the area of the orifice of the diffusion cell. Water vapour concentration (C_i_) inside the fruit/diffusion cell was essentially saturated (19.44 g m^−3^ at 22 °C; [44]), while C_0_ above dry silica gel was practically zero [45]. The experiment was conducted with 12 replications.

### 4.5. Data Analyses and Presentation

Data are presented as means and standard errors, except for individual observations. Where error bars are not visible, they are smaller than the data symbols. Data were analysed using analysis of variance, correlation, and regression analyses. Means were compared using Tukey’s studentised test (*p* ≤ 0.05, package multcomp 1.3-1, procedure glht, R version 3.6.3; R Foundation for Statistical Computing, Vienna, Austria). Regression analyses were carried out using R (version 3.6.3; R Foundation for Statistical Computing) and SigmaPlot (version 12.5; Systat Software, San Jose, CA, USA). SigmaPlot (version 12.5; Systat Software). Significance of coefficients of determination (*r*^2^) at *p* ≤ 0.05, 0.01 and 0.001 are indicated by *, ** and ***, respectively.

## Figures and Tables

**Table 1:** Percent distribution of russet on ‘Apple’ mango fruit along the stem/apex axis on either the blushed or the non-blushed sides of the fruit. Percentage of russet was quantified using image analysis following photography. For the different regions of the fruit, see Figure 7.

Fruit Region	Russeted Area (%)		
	Blushed Side	Non-Blushed Side	Mean Side
Stem end	85.8 ± 6.9	87.2 ± 5.7	86.5 ± 4.4 a ^z^
Basal cheek	47.7 ± 5.7	43.8 ± 5.5	45.8 ± 4.0 b
Apical cheek	19.2 ± 5.8	18.4 ± 5.3	18.8 ± 3.9 c
Apex	15.4 ± 6.1	17.2 ± 6.3	16.3 ± 4.3 c
Mean _Fruit region_	42.0 ± 4.5 a	41.7 ± 4.4 a	

^z^ Main effect but not interaction significant by analysis of variance. Main effect for fruit regions differs according to the Tukey studentised range test, *p* ≤ 0.05. The number of replicates was 18.

**Table 2:** Russeting in ventral and dorsal regions of ‘Apple’ mango from three different sites. The sites were selected because they differed in elevation. Russeting was quantified using a five-score rating scheme. Score 0: 0% of the fruit surface area russeted; score 1: 1–10% russeted area; score 2: 11–25% russeted area; score 3: 26–50% russeted area; and score 4: 51–100% russeted area. For ventral and dorsal regions of the fruit, see Figure 7.

Site	Extent of Russet (Rating Score)		
	Ventral	Dorsal	Mean _Region_
Kaiti	2.9 ± 0.1	3.5 ± 0.1	3.2 ± 0.1 a ^z^
Mumbuni	2.0 ± 0.1	2.8 ± 0.1	2.4 ± 0.1 b
Yeemulwa	1.6 ± 0.1	2.3 ± 0.1	1.9 ± 0.1 c
Mean _Site_	2.2 ± 0.1 b	2.8 ± 0.0 a	

^z^ Main effects “site” and “region” of the fruit were significant at *p* ≤ 0.05. Interaction between site and region of the fruit was not significant in a two factorial ANOVA. Mean separation by Tukey studentised range test, *p* ≤ 0.05. The number of replicates was 200.

**Table 3:** Russeting of peripheral (exposed) or central (shaded) ‘Apple’ mango fruit in the canopy at three different sites. The sites were selected because they differ in elevation. Russeting was quantified using a five-score rating scheme. Score 0: 0% of the fruit surface area russeted; score 1: 1–10% russeted area; score 2: 11–25% russeted area; score 3: 26–50% russeted area; and score 4: 51–100% russeted area.

Site	Russeting (Rating Score)		
	Exposed Fruits	Shaded Fruits	Mean _Fruit Position_
Kaiti	3.7 ± 0.0	3.7 ± 0.0	3.7 ± 0.0 a ^z^
Mumbuni	2.3 ± 0.1	2.5 ± 0.1	2.4 ± 0.1 b
Yeemulwa	2.0 ± 0.1	1.8 ± 0.1	1.9 ± 0.1 c
Mean _Site_	2.7 ± 0.1 a	2.7 ± 0.1 a	

^z^ Main effect of site was significant but neither fruit position nor interaction was significant by analysis of variance at *p* ≤ 0.05. Mean separation according to the Tukey studentised range test, *p* ≤ 0.05. The number of replicates was 200.

**Table 4:** Effect of fruit position within the tree canopy on russeting of ‘Apple’ mango in different sites. Fruit positions were: top (>2 m above the ground), middle (1–2 m), and bottom (0.5–1 m). Russeting was quantified using a five-score rating scheme. Score 0: 0% of the fruit surface area russeted; score 1: 1–10% russeted area; score 2: 11–25% russeted area; score 3: 26–50% russeted area; and score 4: 51–100% russeted area.

Site	Russeting (Rating Score)			
	Top	Middle	Bottom	Mean _Fruit position_
Kaiti	3.9 ± 0.0 a ^z^	3.7 ± 0.0 b	3.5 ± 0.1 c	3.7 ± 0.0
Mumbuni	3.1 ± 0.1 a	2.3 ± 0.1 b	1.9 ± 0.1 c	2.4 ± 0.0
Yeemulwa	2.5 ± 0.1 a	1.7 ± 0.1 b	1.0 ± 0.1 c	1.7 ± 0.0
Mean _Site_	3.0 ± 0.0	2.4 ± 0.0	1.9 ± 0.0	

^z^ Significant interaction between site and fruit position in the canopy in a two factorial ANOVA. Therefore, ANOVA was run by site. Means within the rows followed by the same letter are not significantly different. Mean separation by Tukey studentised range test, *p* ≤ 0.05. The number of replicates was 352.

**Table 5:** Effect of geographical orientation (aspect) of ‘Apple’ mango on russeting. Fruits were sampled from north-, south-, east-, and west-facing sides of the canopy. The tree rows were aligned perpendicularly to the slope and N, S, E, and W positions. Russeting was quantified using a five-score rating scheme. Score 0: 0% of the fruit surface area russeted; score 1: 1–10% russeted area; score 2: 11–25% russeted area; score 3: 26–50% russeted area; and score 4: 51–100% russeted area.

Site	Russeting (Rating Score)				
	North	South	East	West	Mean _Aspect_
Kaiti	3.6 ± 0.0	3.6 ± 0.0	3.8 ± 0.0	3.5 ± 0.1	3.6 ± 0.0 a ^z^
Mumbuni	2.4 ± 0.1	2.5 ± 0.1	2.2 ± 0.1	2.6 ± 0.1	2.4 ± 0.0 b
Yeemulwa	1.8 ± 0.1	1.7 ± 0.1	1.6 ± 0.1	1.8 ± 0.1	1.7 ± 0.0 c
Mean _Site_	2.5 ± 0.1 a	2.6 ± 0.1 a	2.5 ± 0.1 a	2.6 ± 0.1 a	

^z^ Main effect of site was significant but neither aspect of fruit nor interaction were significant by analysis of variance significant at *p* ≤ 0.05. Mean separation according to the Tukey studentised range test, *p* ≤ 0.05. The number of replicates was 264.

**Table 6:** Russeting of ‘Apple’ Mango at ten different sites across Kenya. Russeting was quantified using a five-score rating scheme: score 0: 0% of the fruit surface area russeted; score 1: 1–10% russeted area; score 2: 11–25% russeted area; score 3: 26–50% russeted area; and score 4: 51–100% russeted area.

Site	Maturity (Days after Full Bloom)	Rating (Score)
Thika	196	3.6 ± 0.1 a ^z^
Kaiti	189	3.5 ± 0.1 a
Machakos	226	3.4 ± 0.1 ab
Kasafari	166	3.4 ± 0.1 ab
Chepsigot	146	3.2 ± 0.1 b
Kambirwa	136	3.1 ± 0.1 b
Mumbuni	175	2.3 ± 0.1 c
Yeemulwa	180	1.9 ± 0.1 d
Malindi	113	0.2 ± 0.0 e
Garissa	111	0.1 ± 0.0 e

^z^ Mean separation according to the Tukey studentised range test, *p* ≤ 0.05. Means followed by the same letter are not significantly different. The number of replicates was 210.

**Table 7:** Pearson correlation between climatic variables and russeting for ‘Apple’ mango fruits grown at ten sites throughout Kenya. Climatic variables include rainfall amount, number (n) of rainy days, relative humidity, maximum, minimum, and mean daily temperatures, and heat sum. Heat sum is the sum of mean temperatures above the base temperature of 16 °C. Number of cold nights is the sum of the number of days when the minimum temperature was less than 16 °C. The growth season was divided into three periods of equal duration at each site (early, middle, and late phases). “Cumulative” refers to the entire growth period. Climatic data were obtained from the NASA Langley Research Centre (LaRC) POWER Project.

Weather Parameter	Pearson Coefficients of Correlation (r)			
	Time Period			
	Early	Middle	Late	Cumulative
Rainfall (mm)	0.39 ns ^z^	0.44 ns	0.26 ns	0.60 ns
Rainy days (n)	0.66 *	0.67 *	0.79 **	0.90 ***
Relative humidity (%)	−0.27 ns	0.11 ns	0.08 ns	−0.05 ns
Maximum temperature (°C)	−0.56 ns	−0.71 *	−0.85 **	−0.74 *
Minimum temperature (°C)	−0.94 ***	−0.93 ***	−0.94 ***	−0.94 ***
Mean temperature (°C)	−0.96 ***	−0.95 ***	−0.96 ***	−0.96 ***
Heat sum (°Cd)	−0.57 ns	−0.67 *	−0.72 *	−0.67 *
Dew point temperature (°C)	−0.88 ***	−0.85 **	−0.91 ***	−0.92 ***
Cold nights (n)	0.88 ***	0.73 *	0.63 ns	0.84 **

^z^ Correlation coefficients followed by *, **, and *** were significant at *p* ≤ 0.05, *p* ≤ 0.01, and *p* ≤ 0.001, respectively. Correlation coefficients followed by ns were not significant (*p* > 0.05). The number of fruits inspected at anyone site was 210.
